# Evolvability: filling the explanatory gap between adaptedness and the long-term mathematical conception of fitness

**DOI:** 10.1007/s10539-024-09951-3

**Published:** 2024-07-16

**Authors:** Pierrick Bourrat, Katie Deaven, Cristina Villegas

**Affiliations:** 1 Department of Philosophy, Macquarie University, North Ryde, NSW 2109 Australia; 2 Department of Philosophy and Charles Perkins Centre, The University of Sydney, Sydney, NSW 2006 Australia; 3 Department of Philosophy, University of Wisconsin-Madison, 600 N. Park Street, Madison, WI 53703 USA; 4 Centro de Filosofia das Ciências, Departamento de História e Filosofia das Ciências, Faculdade de Ciências, Universidade de Lisboa, Campo Grande, 1749-016 Lisbon, Portugal

**Keywords:** Fitness, Evolvability, Adaptedness, Natural selection

## Abstract

The new foundation for the propensity interpretation of fitness (PIF), developed by Pence and Ramsey (Br J Philos Sci 64:851–881, 2013), describes fitness as a probability distribution that encompasses all possible daughter populations to which the organism may give rise, including daughter populations in which traits might change and the possible environments that members of the daughter populations might encounter. This long-term definition of fitness is general enough to avoid counterexamples faced by previous mathematical conceptions of PIF. However, there seem to be downsides to its generality: the ecological role of fitness involves describing the degree of adaptedness between an organism and the specific environment it inhabits. When all possible changes in traits and all possible environments that a daughter population may encounter are included in the concept, it becomes difficult to see how fitness can fulfill this role. In this paper, we argue that this is a feature of Pence and Ramsey’s view rather than a bug: long-term fitness accommodates evolvability considerations, which concern the role that variation plays in evolutionary processes. Building on the foundations, we show that Pence and Ramsey’s fitness—*F*—can be partitioned into fourths: adaptedness, robustness of adaptedness, and two facets of evolvability. Conceptualizing these last three components forces us to consider the role played by grains of description of both organisms and the environment when thinking about long-term fitness. They track the possibility that there could be a change in type in a daughter population as a way of responding to environmental challenges, or that the type persists in the face of novel environments. We argue that these components are just as salient as adaptedness for long-term fitness. Together, this decomposition of *F* provides a more accurate picture of the factors involved in long-term evolutionary success.

## Introduction

The notion of evolvability has gained prominence in all branches of evolutionary biology as a way of referring to the potential of biological systems to generate viable variation. Although viable variation is a clear precondition for evolution via natural selection (Lewontin 1985), its production only became a subject of study in recent decades, when it shifted from being a background assumption to an explanatory term (Wagner and Draghi 2010). This was largely due to the realization that biological systems diverge in their capacity to produce variation: while all evolved systems trivially had the potential to evolve the way they did, it is not trivial that they differ in the amount, type, and ways in which they can produce the variation required for evolving.^1^ That is, there are differences in the capacity to generate and maintain variation across traits and clades, and those must be explained in evolutionary terms (Wagner and Draghi 2010).

This idea developed in several domains of evolutionary biology in the 1990s (e.g., Alberch 1991; Houle 1992; Kirschner and Gerhart 1998; Wagner and Altenberg 1996) and quickly became a research agenda for its own sake that fostered dialogue across disciplines (Nuño De La Rosa 2017; Villegas et al. 2023). For example, evolvability has been associated with cryptic variation in population genetics (e.g., Bergman and Siegal 2003; Masel and Trotter 2010); phenotypic response to natural selection in quantitative genetics (e.g., Noble et al. 2019; Hansen and Houle 2008; Holstad et al. 2024); developmental modulations of genetic changes in evo-devo (Hendrikse et al. 2007); and daughter populations’ capacities to generate innovations in macroevolutionary studies (Pigliucci 2008; Wilder and Stanley 2015). Whether evolvability can be unified despite the disparate usages is a thorny question (Brown 2014; Pigliucci 2008; Sterelny 2007; Villegas et al. 2023), due in part to the fact that the explanatory contexts in which it appears study biological systems at different scales: from micro to macro evolution, from genome to clades, etc.

The difficulties of characterizing evolvability across scales and domains are reminiscent of the problem of characterizing fitness. Fitness is a—if not *the*—central concept in evolutionary biology. Applied broadly, it relates properties of biological entities at any scale and in any domains to evolutionary success. However, evolutionary biologists have had a difficult time defining fitness, and there is much disagreement across disciplinary approaches regarding how the concept should be applied (Abrams 2012; Ariew and Lewontin 2004; Hansen 2017; Michod 1999, Chapter 8; Stearns 1976). In the philosophy of biology, this situation has famously generated a whole range of positions on how best to define fitness, both in terms of what kind of content the concept has (e.g., causal, explanatory, statistical, predictive) and in terms of which entities it must be predicated of (e.g., genotypes, organisms, traits, populations).

However, the links between fitness and evolvability go beyond this similarity. Fitness and evolvability are both recognized sources of evolutionary success. Fitness is often characterized in terms of survival and reproductive output (Brandon 1978, 1990; Mills and Beatty 1979; Sober 2001), or growth rate of a trait within a population (Walsh, Ariew, and Matthen 2017, Takacs and Bourrat 2022, 2024). Evolvability, on the other hand, is characterized in terms of the production and maintenance of the heritable variation that determines the set of evolutionary trajectories available to a population (Brown 2014; Love 2003; Ramsey and Villegas 2024; Sterelny 2007). While both concepts are cited in explanations of differential evolutionary success, the nature of the relationship between the two remains unclear. One may presume that they interact synergistically and therefore question whether the two notions may be somewhat redundant. For example, do differences in fitness amount to differences in evolvability and vice versa? Proponents of evolvability across biology and philosophy use this notion as non-overlapping with fitness but recognize that they are connected in some way. One focal point within the evolvability literature aims to address the ways in which evolvability may enhance fitness and whether evolvability, itself, is an adaptation (Pigliucci 2008; Sniegowski and Murphy 2006; Wagner 2022, 2023; Wagner and Draghi 2010; Wagner and Altenberg 1996). However, one might see evolvability and fitness as being at odds with each other, appealing to cases where increases in evolvability seem to involve fitness costs (Chou et al. 2011; Gibson et al. 2017; Smith 1971). These discussions suggest that the two concepts play different explanatory roles as well as highlighting a lingering lack of clarity about their exact interactions and relative contributions to evolutionary success.

In this paper, we propose a philosophical framework to understand the relationship between evolvability and fitness. In particular, we provide an account that reconciles cases where it seems like evolvability and fitness clash and explains why, in others, they seem to complement each other. We argue that these apparent conflicts can be resolved if it is recognized that ‘fitness’ sometimes refers to the notion of adaptedness to the immediate environment of an organism and sometimes is associated with long-term evolution predictions that far exceed the lifespan of an organism. In particular, our framework shows that evolvability can be understood as a component of long-term fitness, but also that this does not make the concept of evolvability redundant. Starting from the most sophisticated propensity interpretation of fitness (PIF) as a measure of long-term evolutionary success (Pence and Ramsey 2013), we show that long-term fitness decomposes into four components, two of which ought to be interpreted as forms of evolvability. Key to this decomposition are the notions of the ‘environment of reference’ and ‘transmission of type’ (or form) over time.

We first review the classical debate on adaptedness and the PIF (Section “The classical PIF and its problems”), as well as Pence and Ramsey’s new PIF proposal as a measure of long-term fitness (Section “The new PIF”). Then, we discuss several problem cases regarding long-term fitness as capturing adaptedness simpliciter (Section “Problem cases for interpreting the new PIF as adaptedness”). We then introduce our proposal of fine-graining long-term fitness by partitioning it into four components: adaptedness, robustness of adaptedness, and two kinds of evolvability (Section “Decomposing *F*: Bridging the new PIF and evolvability”). Finally, we discuss the advantages of our proposal in the context of several controversies in the philosophy of evolution and dispel several potential objections to it (Section “Putting *F* into context”).

## The classical PIF and its problems

Characterized as the ‘two faces of fitness’ (Sober 2001), fitness plays two roles in evolutionary theory. As an *ecological descriptor*, fitness refers to the degree of adaptedness between an organism and its specific environmental conditions. As a *mathematical predictor*, fitness is often characterized as a scalar numerical value that can be used to produce a rank ordering of organisms based on these values and, consequently, justify ecological claims like ‘*a* is fitter than *b*’ and predict changes in trait frequencies.^2^

Bridging these roles has proven a difficult task that is plagued with problems. The most famous of these is the so-called ‘tautological problem.’ If fitness is defined in terms of realized reproductive success, it cannot be explanatory, since the claim that ‘organisms that have higher fitness leave more offspring’ amounts to ‘organisms that leave more offspring are those that leave more offspring.’ The PIF provides a solution to this problem by holding that an organism’s fitness is its *disposition* to produce offspring (Beatty and Finsen 1989; Brandon 1978; Mills and Beatty 1979; ﻿Villegas and Morales Carbonell 2024). On this account, fitness causally explains why an organism with a higher fitness will tend to be reproductively successful, even if its realized reproductive output is low. Thus, the PIF provides an account of fitness that fulfills the role of an ecological descriptor, therefore referring to adaptedness. However, the PIF is not without problems. This disposition, once characterized formally, describes a probability distribution over the number of offspring the organism might produce with differing probabilities assigned to each possible number. PIF proponents naturally argued that an organism’s fitness should, therefore, be defined as the expected number of offspring. However, this traditional formulation falls prey to a number of problems and generates a repository of counterexamples.

To cite one well-worn example, Gillespie (1974) argued that the mean value of the probability distribution alone can be insufficient to predict the evolutionary success of a type. When the environment varies periodically, a lower variance in reproductive output will also be a predictor of evolutionary success. Following Gillespie’s work, Beatty and Finsen (née Mills) (1989) refined their PIF account by including other moments of the probability distribution (e.g., variance and skew), persuaded that ignoring these features results in inaccurate predictions regarding trait frequency changes. Since then, several more charges have been laid out. First, the expected change in trait frequencies from one generation to the next may not be a suitable predictor of long-term evolutionary success, for reasons other than not taking into account the effect of other moments of the probability distribution. As Ahmed and Hodgkin (2000) have shown, mutations resulting in sterility may not be expressed for many generations, which risks arbitrary assessments of fitness.^3^ Further, timing of reproduction can swamp judgments about fitness; *ceteris paribus*, if two organisms have the same number of expected offspring but one has a shorter generation time, that one will be fitter (Pence and Ramsey 2013).^4^

Faced with these problems, contributors to this literature have split into roughly three camps. Some have adopted the statisticalist interpretation of fitness, treating trait fitness^5^ as a statistical summary, divorced from considerations based on organismal dispositions (Matthen and Ariew 2002; Walsh, Ariew, and Matthen 2017). Others have explored different causal interpretations of fitness, natural selection, and drift that do not rely on the PIF (e.g., Abrams 2007, 2012, 2023; Bouchard and Rosenberg 2004; Bourrat 2017; Godfrey-Smith 2009; Otsuka 2016). Still others propose that PIF generates not one but a family of propensities and corresponding fitness measures (Beatty and Finsen 1989; Brandon 1990). The aim of this third camp is to reconcile the two roles of fitness within the PIF framework, either by introducing corrective measures to address counterexamples (Brandon 1990) or proposing new foundations for PIF (Pence and Ramsey 2013; Ramsey 2006). This third camp is the focus of this paper.

As Pence and Ramsey (2013) note, adding corrective measures is not promising, as it does little to convince the statisticalists that the PIF framework is needed. Further, there is no single corrective measure that can handle all of the counterexamples that have been identified. The real problem does not touch the PIF thesis—the fitness of an organism is the propensity of an organism to produce offspring—but constitutes a challenge to how fitness should be measured. The promise of the new foundation for PIF is that it will provide a measure of fitness that may be applied in every evolutionary system and is impervious to the kinds of counterexamples outlined above. In other words, this new foundation is an attempt to solve what Pence and Ramsey call the ‘generality problem.’ We see the merits of the PIF and now turn to considering the extent to which the new foundation succeeds in reconciling the two faces of fitness.

## The new PIF

Pence and Ramsey’s new foundation for PIF is inspired by Ramsey’s (2006) ‘block fitness’ account, which defines an organism’s fitness in the infinite long run. Ramsey (2006, 2016) describes block fitness as a fixed quantity derived from the structure of what he calls the possible life histories of an organism. Building on the PIF foundation, Ramsey maintains that an organism’s fitness is a fixed (time-invariant) property that encompasses all possible life histories of the organism—that is, all the ways in which its life can unfold. He regards his account as compatible with the classical PIF (2006, p. 485).

Building on this insight, Pence and Ramsey (2013) propose a measure that describes an organism’s fitness as a probability distribution encompassing all potential daughter populations to which the organism may give rise. Each daughter population is weighted by the associated probability of being the actual daughter population of the organism. Taking into account the sizes of all the possible daughter populations, the fitness of an organism is represented by a scalar value, *F*. This definition of fitness is promising, as it resists the kinds of counterexamples faced by the classical PIF framework (Pence and Ramsey 2013). The new formulation can take the form of a geometric mean to account for higher moments of the distribution when needed. Further, it is an asymptotic measure that accommodates changes in expected descendants across generations. Finally, this definition determines fitness based on absolute time rather than generations. This means it can also resolve the timing of reproduction counterexample, so long as the timescale is at least as long as the longest generation time. Since Pence and Ramsey define their measure of fitness based on an infinite limit, this is guaranteed.^6^

We laud Pence and Ramsey for proposing this general definition; the new foundation for PIF provides a strong basis for philosophical discussions of the concept of fitness, as well as a compelling answer to the generality problem. Further, this new foundation is built on a measure of fitness used in adaptive dynamics, an influential field in evolutionary biology. However, there are potential challenges that arise from theorizing about fitness at this level of generality. Recall that the ecological role of fitness involves describing the degree of adaptedness between an organism and the specific environment it inhabits. When all possible changes in phenotype that might evolve within the daughter population and all possible environments are included in the concept, it becomes difficult to see how *F* can fulfill a role that depends on the relationship between an organism with a particular set of traits (a reference type) and a particular environment (a reference environment) classically associated with adaptedness. It follows from their account that *F* depends on an infinite number of possible combinations of specific descendant lineages, where descendants can differ in type from the ancestor (the referent organism) and encounter an infinite range of environmental settings. We suggest that this poses a challenge to the alleged translation between adaptedness and fitness as a long-term measure, an undesirable feature for Pence and Ramsey and others committed to revising the PIF. To see this, consider an ancient aquatic organism. Given all the daughter populations it might give rise to in the long run—including some of which having the phenotype ‘presence of wings’—in all the possible environments—including terrestrial ones—yields a concept of adaptedness that seems far removed from the classical idea of an organism’s fit with its environment, as conceived by Brandon (1990).^7^ This is so because the type and environmental conditions against which adaptedness is assessed become too unspecific. However, we argue that the separation of long-term fitness and adaptedness is a feature of Pence and Ramsey’s view rather than a bug because it accommodates the multifaceted nature of fitness in the long-term. We motivate this line of reasoning by considering four very different examples of what fitness in the long term might look like.

## Problem cases for interpreting the new PIF as adaptedness

One aim of Pence and Ramsey’s project is to provide an account of fitness in the spirit of PIF, where organismal fitness (i.e., adaptedness) is fundamental, while at the same time solving the generality problem by providing a definition of fitness that correctly tracks evolutionary success for any biological entity in question. However, as we have hinted with the example of an ancient aquatic organism, this mathematical formulation of fitness deviates from the classical idea of adaptedness. We argue that by proposing that fitness describes a probability distribution of all possible daughter populations (including arbitrarily distant daughter populations with significantly different features) and in all possible environments that these might encounter, Pence and Ramsey cannot maintain the link between adaptedness, as traditionally conceived, and long-term evolutionary success. To illustrate this, we present four hypothetical cases, each of which compares two organisms where one would have a higher long-term fitness *F*, following Pence and Ramsey’s proposal. However, we argue that only the first of these cases corresponds neatly to a case where the adaptedness of the two organisms differ.

The first case compares two organisms, where all of the possible daughter populations^8^ to which each organism can give rise are of the same type as the ancestor and the environment stays the same through time; in other words, all members of the possible daughter populations have the reference type and live in the reference environment. The canonical case of dark and light moths (Kettlewell 1955) serves to illustrate this possibility, supposing that the post industrial environment persisted indefinitely. In a polluted environment, a dark moth has higher adaptedness than a light moth due to a higher chance of camouflaging in darkened woods. Provided that the environment never changes and the lineages to which these moths give rise never change their phenotypes, it follows that the dark moth has a higher *F*, as established by the new PIF. In this case, *F* would be satisfactorily explained by the classical idea of adaptedness in the following way: since all daughter populations of each organism will preserve the same relevant relations with the environment, their ecological adaptedness is responsible for the long-term success of its correspondent descendant lineages and, therefore, completely grounds its mathematical fitness.

The second case compares two organisms where all of the possible daughter populations to which each gives rise are of the same type as the ancestor, but the environments that the members of the daughter populations would encounter are different from the environment of reference. The so-called ‘living fossils’ taxa, composed of similar members now and in the deep past, can help exemplify this case. Consider two organisms living in a population of ancestral rynocephalians during the early Mesozoic-era. The first organism is the ancestor of the tuataras, an extant species of reptile found in New Zealand that is considered a living fossil (Herrera-Flores et al. 2017). The second organism is the ancestor of the closely related extinct species *Opisthiamimus gregori*, which had similar morphology to its common ancestor and that of the tuatara (DeMar et al. 2022). Suppose that both of these ancestral organisms were equally well adapted to the Mesozoic environment. What explains the stark difference in the evolutionary trajectories of their descendant populations? Perhaps the reason for this is that the ancestor of tuataras had generalist features, whereas the ancestor of *Opisthiamimus gregori* had specialist features, resulting in the former’s offspring being able to survive changes in environmental conditions that the offspring of the latter could not. Importantly, *F* tells us they are not equally fit, even with the stipulation that the ancestral organisms were equally well adapted in their environment and the members of their respective daughter populations inherit the reference type. We think that this highlights another feature of fitness to which *F* is sensitive: what distinguishes the two outcomes is how *robust* the adaptedness of each organism is to changes in the environment of reference. Thus, in this case, the robustness of the ancestor of tuatara’s adaptedness is higher than that of the ancestor of *Opisthiamimus gregori*.

The third case compares two organisms, where some of the daughter populations to which the organisms give rise are of a different type than the ancestors, but the environment remains the same as the environment of reference. As an example of this case, consider a population of snakes that is threatened by an endemic pathogen. Suppose that while our first snake reproduces asexually, the second is able to reproduce sexually, and that changes in type that make the organisms more resistant to this disease occur via mutations in each of their possible daughter populations at the same rate. One might expect that *F* will be equal for both snakes, if the same new mutations arise in the same number of possible daughter populations for both types.^9^ However, this would be a hasty conclusion. Because our second organism of interest reproduces sexually, its realized daughter population is much better disposed to resist the pathogen than the realized daughter population of the snake that reproduces asexually. This is so because beneficial mutations from two sexually reproducing organisms can be combined to produce offspring that are even more resistant (assuming an additive effect of mutations), while they cannot in strictly asexual organisms (Maynard Smith 1978, 1989; Muller 1932). Observe that, in this case, the adaptedness of the ancestral snakes cannot explain why *F* will be higher for the sexually reproducing snake than for the asexually reproducing one: both snakes are just as poorly adapted to the environment of reference. Here, the organism’s ability to produce offspring that are different from it and more adapted to the environment is also a determinant of *F*. However, this is not only true when organisms are poorly adapted to their environment of reference. It will rarely be the case that an environment remains the same indefinitely. Even when organisms are well adapted to the environment of reference, as we will show in our final case, this sort of concern remains an important consideration for calculating *F*.

Our fourth case compares two organisms where some of the daughter populations to which they give rise are of a different type than the referent organisms, and the environments encountered by members of these possible daughter populations are different from the environment of reference. To illustrate this fourth case, consider two bears who both have long fur, making them equally well adapted to the cold climate they live in (Orzack and Sober 2001). Suppose, however, that our first bear has a genetic architecture that would allow it to acquire a mutation^10^ for short fur without incurring any deleterious side effects, while for the second bear, a change in fur length would result in at least one significant deleterious effect. By considering the daughter populations that encounter warm environments, we can see a striking difference in the values of *F* for the two ancestral bears. In these environments, the offspring of the first bear—assuming inheritance of the genetic architecture—will be far better adapted than the offspring of the second one who cannot develop the short fur without incurring costs. Again, we see that adaptedness does not translate to high values of *F*: both bears are equally well adapted to the environment of reference, but the daughter population of the first bear will be much more likely to persist than that of the second bear. Thus, it is not only the ability to change type in response to environmental challenges within the environment of reference but also the ability to change type in response to challenges in new environments that will contribute to high long-term fitness. This case illustrates a counterpart to our second case, involving the ancestor of the tuatara; a high value of *F* might be achieved when the referent type assumes a ‘generalist’ strategy, but organisms that are ‘specialists’ *and* able to change in the face of new environments could also have high long-term fitness.

These hypothetical cases show that using *F* helps us to identify when one lineage is more evolutionarily successful than another more accurately than using adaptedness alone. In all these cases, we have explicitly assumed that the two organisms have the exact same adaptedness, yet seen that the long-term fitness of the organisms may have very different values. Indeed, Pence and Ramsey (2013) do not see any tension between adaptedness and long-term fitness (pp. 852, 853, 868). However, our three final cases show that the features of robustness (of adaptedness) and (what we will identify as two aspects of) evolvability do not correspond to adaptedness simpliciter but are nevertheless crucial to measuring long-term fitness accurately.^11^ This is a yet unnoticed, but attractive, feature of their view. In the next section, we show how *F* may be partitioned into these components and outline the theoretical implications of this partitioning.

## Decomposing *F*: Bridging the new PIF and evolvability

**Fig. 1:**
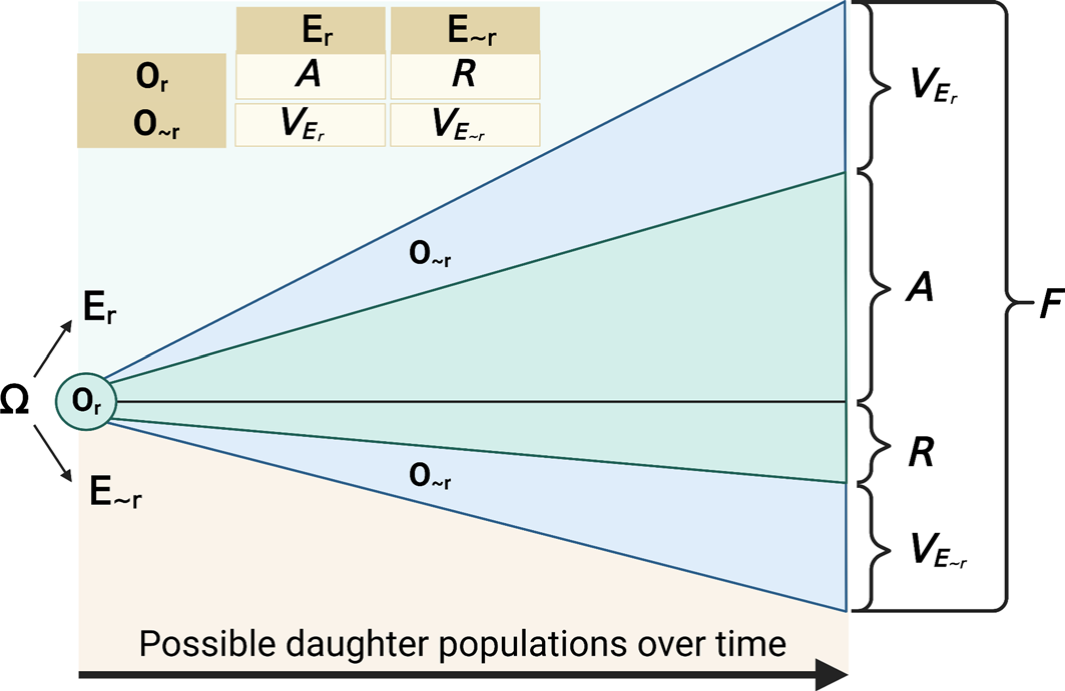
An organism of reference ($O_{r}$)—that defines a type of reference—has multiple possible lives in which it produces distinct daughter populations, weighted by the probability of each event occurring during the life of the organism and that of its descendants. Altogether, this characterizes *F*, the total fitness of the organism, as proposed by Pence and Ramsey (2013). The higher the number of descendant organisms constituting the possible daughter populations, weighted by their probability of being the actual daughter population, the larger the fitness of $O_{r}$. We propose a decomposition of *F* into four components, with each triangle referring to one partition. The partitions are defined by whether the members of the possible daughter population belong to the same type as $O_{r}$ and whether the members encounter the environment of reference $E_{r}$ or a novel environment $E_{ \sim r}$. Together, $E_{r}$ and $E_{ \sim r}$ represent the set of all possible environments Ω, weighted by the probability that a particular environment is the actual environment. Adaptedness *A* is the component of *F* that refers to daughter populations of the same type as $O_{r}$ living in the same environment as $E_{r}$. Robustness (of adaptedness) (*R*) is the component of *F* that refers to daughter populations of the same type as $O_{r}$ but in a different environment from $E_{r}$. Evolvability *V* is the component of *F* that refers to daughter populations of a different type as $O_{r}$. We further decompose *V* into two subcomponents, depending on whether the members of the possible daughter population would encounter the environment of reference $E_{r}$ ($V_{{E_{r} }}$) or not ($V_{{E_{ \sim r} }}$). We have simplified the figure here because it would be possible, for instance, that a descendant member of the population that is of the same type as $O_{r}$ in the environment of reference produces offspring of a different type and/or who will encounter a different environment from $E_{r}$. In such cases, the adaptedness of $O_{r}$ should be discounted from all the subpopulations of descendants that are of a different type as $O_{r}$ and in a different environment from $E_{r}$. Mutatis mutandis, the same applies to the other components of *F*. We have assumed here, for illustrative purposes, that daughter populations retain the type and evolve in the same environment as the direct offspring of $O_{r}$ indefinitely

As we have shown in Section “Problem cases for interpreting the new PIF as adaptedness”, *F* corresponds neatly to adaptedness only when the offspring or more remote descendants have the same type as the focal organism and the environment of reference stays the same indefinitely. We think the other three cases motivate a way of partitioning *F* into fourths: (1) daughter populations where the type of the descendants and environment are the same as the referents, like dark moths in polluted environments; (2) daughter populations where the type of descendants is the same type as the referent organism but the environment changes, like the Mesozoic ancestor of the tuatara, whose offspring roughly retain its features throughout the environmental changes that have occurred in the last 250 million years; (3) daughter populations where the type of the descendants is different from the type of the referent organism but the environment stays the same, like the sexually reproducing snakes who are able to survive within a threatening environment; and (4) daughter populations where the type of the descendants is different from the type of the referent organism and the environment also changes, like our first bear that can develop a short coat that makes it better suited for the warming of its environment without deleterious side effects. We represent this decomposition in Fig. 1 and describe each partition of *F* below the figure.

*F* is the measure of how well an entity (typically an organism) $O_{r}$ is able to produce descent, irrespective of the environment they could encounter and how different from the original type they are. By decomposing *F* by whether possible daughter populations are of the same type as $O_{r}$ and in the same environment as the environment it encountered ($E_{r}$), we can appreciate the properties, other than adaptedness, that are relevant to *F* and establish links between them. In particular, we propose that four different properties, with adaptedness being only one of them, each map to one portion of the probability distribution of $O_{r}$’s possible daughter populations:

- Adaptedness (*A*), which refers to the portion of $O_{r}$’s possible daughter population of the same type in the same environment as $O_{r}$. It measures how well $O_{r}$ is able to survive and produce descendants of the same type that reproduce in a particular environment ($E_{r}$).
- Robustness (of adaptedness) (*R*), which refers to the portion of $O_{r}$’s possible daughter populations of the same type as $O_{r}$ but in a different environment $E_{ \sim r}$^12^ It measures how well $O_{r}$ would be able to survive and produce descent that is of the same type and adapted to an environment different from the environment of reference ($V_{{E_{ \sim r} }}$).
- Evolvability (*V*), which refers to the portion of $O_{r}$’s possible daughter populations of a different type from $O_{r}$. It measures how well an entity is able to change its type over evolutionary time^13^ and/or produce descent that is different from it and adapted to the environment it encounters. We decompose *V* into two subcomponents: $V_{{E_{r} }}$, which measures evolvability in the environment of reference, and $V_{{E_{ \sim r} }}$, which measures it in a different environment.

According to our decomposition, adaptedness only refers to the part of evolutionary success that can be ascribed to a referent type in its environment of reference, while robustness (of adaptedness) can be seen as a measure of how well the referent type would do in a different environmental situation, which involves some extrapolation of the entity’s adaptedness. Finally, evolvability considers the entity’s capacity to produce descent that differs in type and how well the descent would do in the environment of reference ($V_{{E_{r} }}$) or in a different environment ($V_{{E_{ \sim r} }}$), given its change in type. While the concept of evolvability is defined in many ways (see Section “Putting *F* into context” for further discussion), we believe that our interpretation of evolvability preserves the core features of the concept as seen in the evolvability literature—namely, the capacity to produce and maintain new variation. As we have shown, the manifestation of this capacity plays a key role in understanding the different ways in which a biological entity may be relatively fitter than others in the long term. Further, our decomposition shows that generally, unless one considers that the reference type is defined solely by filiation and that the environment of reference includes all possible environments (more on this point in Section “Putting *F* into context” as well), an organism’s long-term fitness cannot be accurately described by just one of these components. Rather, it will always consist of some particular combination of the four of them.

Changes in type, measured by $V_{{E_{r} }}$ and $V_{{E_{ \sim r} }}$, are prima facie compatible with Pence and Ramsey’s view. They anticipate a potential objection along this line, noting that organisms included in the daughter population might be very different from their ancestor. While it may seem strange for an ancestor’s fitness to be dependent on its far-removed descendant, and precise measurements of fitness may be inaccessible in these cases, Pence and Ramsey consider that these two general problems for measuring fitness accurately are not specific to their account (Pence and Ramsey 2013, p. 870). Further, providing a general definition of long-term fitness should be distinguished from operationalizing it into empirically tractable measures (Beatty and Finsen 1989; Millstein 2014). In their view, similarity of descent is not relevant for long-term organismal fitness (unlike for long-term *trait* fitness). Here, it is the size of the daughter population that matters, and being a daughter population of an organism is a binary relation that does not come in degrees: you either are a descendant of an organism or you are not, no matter how distant in time or in phenotype you are from it (Pence and Ramset 2013, p. 870).

However, we think there is more to be said on this point if our mathematized notion of fitness is supposed to be transcribable from adaptedness as an ecological descriptor, as the PIF generally intends. Remote descent might be very different from their ancestors, and defining the ‘fitness of an organism’ in such a way that it includes information about descendants that are very different from the ancestral organism loses some of the insights captured by ‘adaptedness.’ These implications result in an explanatory gap between the idea of adaptedness and the notion of fitness and might lead one to think that accepting Pence and Ramsey’s view results in a loss of explanatory power for the concept of fitness (or a departure from the kind of account of fitness that earlier PIF proponents have sought).

Our claim is that the notion of adaptedness on its own is insufficient to capture the nature of long-term fitness. To make sense of long-term fitness, one must move away from the notion of adaptedness, as it is classically conceived, and take into consideration factors that relate to environmental changes and changes in (pheno)types. Indeed, hints of this thinking can be seen by examining the predecessor of Pence and Ramsey’s view—the idea of block fitness, as formulated by Ramsey (2006). Block fitness is close in spirit to Brandon’s adaptedness, since it concerns the possible ways in which organisms could interact with their environment within their lifetime.

Nonetheless, in mathematizing the block fitness idea and introducing the idea of distant daughter populations—an idea Ramsey (2006, p. 495) was initially skeptical about—Pence and Ramsey have added several additional features. We contend that these features involve changes in the type and environment of reference, which has implications for *F* as a generalization of the PIF. These factors are captured with our three additional components of *F*.^14^

In sum, the fact that the possible type-environment combinations included in *F* are so diverse suggests that the formulation is something more than a sophisticated generalization of the classical notion of adaptedness. Since Pence and Ramsey (2013) aim to construct a new mathematical foundation that is continuous with the historical conceptions of PIF, including Brandon’s idea of adaptedness (1990), the fact that additional properties like evolvability and robustness must be considered to make sense of it should be made explicit. However, we posit that this is not inherently problematic. Before responding to some potential objections to our proposal, we delineate several strengths of our partition, especially when seen as a fruitful extension to, rather than criticism of, Pence and Ramsey’s new PIF.

## Putting *F* into context

There are several advantages to our proposal. The first is that it inherits all the solutions to the problems solved by Pence and Ramsey’s *F*.^15^ This is so because our account constitutes a partitioning of *F* into four components and, consequently, contradicts neither Pence and Ramsey’s results nor the powerful mathematical foundations it is built upon in any way. Our contention is focused on the way *F* should be *interpreted*. On this front, it has an advantage over Pence and Ramsey’s interpretation of *F* in that it clarifies in what sense *F* relates to the classical PIF, the latter of which is primarily concerned with the notion of adaptedness. It does so by recognizing that the concept of adaptedness is coupled with a particular environment (which might refer to multiple states) and a particular phenotype (which might be a pattern of phenotypic expression or norm of reaction). All possible descendant populations that do not conform to this environment and referent type leave an explanatory gap between the mathematical conception of fitness, *F*, as envisaged by Pence and Ramsey, and the classical PIF. Following our account, we interpret *F* as a much more general measure of fitness, one that accounts for situations in which either or both the environment of reference and type of reference can change. When this occurs, one must invoke different explanatory resources. This yields another benefit—namely, that the relationship between fitness, two facets of evolvability, and what we call robustness of adaptedness become unified under Pence and Ramsey’s framework.

### Unificatory and explanatory power of our decomposition

Our framework permits the drawing of interesting links across different literatures. First, our quadripartite decomposition brings together the literatures on fitness and on evolvability. We suspect that one reason why these literatures^16^ have not interacted more in philosophical discussions is that the organisms comprising a population usually have the same evolvability-related features. Differences in developmental properties, mutation rates, trait covariation patterns, pleiotropic effects, or reproductive strategies (to name a few) are far more visible across species than in intra-population comparisons,^17^ and it is the latter that typically concern philosophical discussions on fitness. However, we see no tension here. Although competition between two types within a population will typically not involve evolvability-related property differences, the latter becomes essential when considering long-term fitness, regardless of intra-population variation comparisons.

Our quadripartite distinction is not solely relevant for hypothetical cases such as the one presented in the third section. It can be useful to understand the different explanatory roles that the notions of fitness as adaptedness, of robustness, and of evolvability play in evolutionary research. This is most evident in cases in which there are apparent conflicts between these concepts, which we highlighted in the introduction. Studies have shown that some neutral, or even slightly deleterious, changes can increase prospects of long-term evolutionary success by augmenting the capacity to generate new adaptive variation. For example, the accumulation of cryptic variation in robust systems enables further exploration of the adaptive landscape, thus facilitating the acquisition of new adaptive phenotypes (Wagner 2022, 2023). We interpret this to be a case in which long-term fitness is enhanced by an increase in evolvability without affecting adaptedness or even at the expense of it. This point can also be seen following Wilkins and Godfrey-Smith (2009; see also Ramsey and Villegas 2024) by considering that adaptedness is important for explaining adaptations to *local* peaks, but insufficient for explaining how populations move across valleys on the adaptive landscape.

Another example where fitness, as conceived by the classical notion of adaptedness, and evolvability seem to clash comes from experimental evolution studies in bacteria. Here, researchers found that the epistatic interactions between beneficial mutations (e.g., mutations that increase adaptedness) decelerate the rate of evolutionary *adaptive change* (Bruggeman et al. 2023; Chou et al. 2011). We interpret this to be a case in which an increase in adaptedness indirectly decreases evolvability. Observe that such a loss does not mean the lineage will go extinct. The loss of evolvability that is incurred need not be a problem if the environment remains stable enough. On the other hand, our quadripartite decomposition also helps to enlighten historical conflicts, like those surrounding the evolution of sexual reproduction. The evolution of sex was particularly perplexing since sexual reproduction involves a short-term fitness cost due to an investment in males and the loss of fidelity of inheritance wrought by meiosis (Gibson et al. 2017). The key to explaining the evolution of sexual reproduction and ubiquity across the tree of life is the long-term benefit of recombination, which enhances the entity’s capacity to respond to changing conditions (Kondrashov 1988). We interpret this as a case where a loss of adaptedness is compensated by an important increase in evolvability.

Our framework also serves as a bridge between evolutionary and ecological research. Two notions that provide interesting links are ecological resilience (e.g., Delettre 2021; Gunderson 2000) and the concept of mismatch in evolutionary medicine (see Bourrat and Griffiths 2021; Griffiths and Bourrat 2023). Both—a mismatched organism and a less resilient ecosystem—do worse in a new environment than in their environment of reference. In the case of mismatched organisms, they might have a low *F* not because they had a low adaptedness in their ancestral environment, but due to a low robustness of their adaptedness. Our framework also permits us to draw parallels with discussions in evolutionary ecological studies that deal with the potential adaptive responses of organisms to climate change. Reframed in our terms, they illustrate the importance of considering robustness of adaptedness and evolvability when making evolutionary predictions. In these, both the capacity of a particular type to survive in a different environment as well as its capacity to produce changes—often referred to as evolvability—are important for predicting evolutionary changes (e.g., Gienapp et al. 2017).

### Tidying up our decomposition

Our quadripartite decomposition inherits the solutions that plagued earlier accounts of the PIF, provides a way of interpreting Pence and Ramsey’s new foundation that bolsters their claims that the new foundation is rooted in the classical PIF tradition, and unifies literatures that have, thus far, not interacted as much as they could. Despite its application to real-world cases shown in Section “Unificatory and explanatory power of our decomposition”, the theoretical nature of our proposal may give rise to some reservations about how it may be used in practice. We anticipate two main objections. One objection is that the two features that ground our decomposition—the environment and type of reference—are arbitrarily defined, and, consequently, the benefits of our framework count for very little. The second objection is the worry that the terms we attribute to our four components, especially those that pertain to evolvability, do not correspond to what has traditionally been associated with them.

Regarding the first objection, what is considered the type of reference and the environment of reference can indeed be arbitrary according to our proposal, in the sense that different researchers within a field or, more likely, between different fields might consider different points of reference for the biological objects they study. Nevertheless, we do not consider this to be a bug but rather a feature of our account. We regard our proposal as a general explanatory scheme that can be applied across different scales and include different contexts. What, at one scale, is a change of type can be regarded as part of the plasticity of the type from a different perspective (see fn 13). Thus, how much within-type genetic, phenotypic, and environmental variation one allows for is a matter of explanatory relevance. Further, as argued by Bourrat and colleagues in several places (see Bourrat 2020; Bourrat and Charbonneau 2022; Charbonneau and Bourrat 2021; Takacs and Bourrat 2021), evolutionary theory, in and of itself, does not have the resources to tell us at what grain of description a particular evolutionary explanation should be used. Instead, the relevant grain is fixed by a community of researchers. This last point is reminiscent of Griesemer’s warnings (2005) against what he calls ‘strategies for generalization-by-abstractions’ encountered in evolutionary theory.^18^

Perhaps Pence and Ramsey (or those endorsing their framework) may argue that the environment of reference and the type of reference should be maximally broad, so as to include all possible types and all possible environments.^19^ In principle, we see no problem with this strategy. However, in doing so, as we have argued, the link with adaptedness is lost. While Pence and Ramsey introduce all possible descendant lineages and environments to make the PIF suitable for long-term evolution rather than just for the short term—which was Brandon’s (1990) original idea— a discussion of how that *F* as a concept is missing from their account. Perhaps one might maintain that, in the broadest sense, ‘adaptedness’ includes information about the potential to change (so as to be adapted to an environment varying widely: say, from aquatic to terrestrial). However, as was already mentioned, this would be a stretch since this notion of adaptedness would be quite different from the one the proponents of the PIF had in mind. We think the better move is to follow the early PIF proponents in recognizing that adaptedness refers to a particular organism in its environment, which our view neatly accommodates.

Further, by recognizing that there is no, in principle, privileged grain at which to describe an evolutionary trajectory, we can see why different fields achieve little consensus regarding the way to interpret a particular phenomenon, despite using the same explanatory scheme (i.e., the Darwinian theory of evolution). We think that at least part of the answer lies in the fact that these fields use different grains of description in applying the terms of the scheme and are referring to different objects (or the same objects) in different contexts or over different scales. By providing placeholders for the type and environment of reference to measure long-term fitness, our proposal invites practitioners to explicate the level of description or grain they are using.

To the second objection—that our components do not correspond to the terms used in the literature—we respond that, at an abstract level, our decomposition relies on the basic separation of evolutionary change into two components. The first concerns changes in the *distribution* of the (type of) objects forming a population—such as selection—while the other concerns changes that are due to the *transformation* of the objects forming a population—such as (but not limited to) mutation. This way of partitioning evolutionary change has been proposed by different authors (e.g., Endler 1986; Godfrey-Smith 2009; Lewontin 1983) and is used in general equations for predicting evolutionary change such as the Price equation (Price 1970)^20^. The separation of transformational aspects of evolution from the distributive (more particularly, selective) ones has been a major goal of the main protagonists involved in developing a research program on evolvability (Nuño de la Rosa 2023). Our framework aligns with this separation, integrating both aspects of evolution into a long-term conception of fitness.

In addition, distinguishing capacities in an environment of reference from those relevant in a different environment accommodates two important aspects of evolutionary success. On the one hand, it considers the *robustness of adaptedness* as a key component of *F*, which allows for identifying cases of ecological resilience and evolutionary mismatches described above. On the other hand, it distinguishes two components of evolvability that refer to two of its facets in the biological literature. $V_{{E_{r} }}$ resonates with the idea that evolvability is crucial for generating complex adaptations to specific environmental challenges (Wagner and Altenberg 1996). To see this, notice that the sampling of variants that selection entails fails, in and of itself, to generate new variation without which complex adaptations cannot be produced. Evolvability, seen as the capacity to produce new variation (e.g., through new mutations and the recombination of alleles), is therefore an important component of adaptive evolution. In addition, $V_{{E_{ \sim r} }}$ resonates with the idea that accumulated cryptic variation can enhance evolvability because it can be released in new environments where it can provide different adaptive solutions (Wagner 2023).

However, perhaps this objection cuts deeper than we have, thus far, considered. According to our framework, evolvability refers to those factors *producing and maintaining new variants*. As we described earlier, we think this captures the core meaning of evolvability across the literature (Villegas et al. 2023). However, we would be remiss not to acknowledge the myriad of definitions of evolvability. Theorists disagree about the bearers of evolvability, the physical bases that contribute to the bearer’s evolvability, and what evolvability is said to explain (Brigandt et al. 2023). Indeed, varying uses of the term (and corresponding ways in which it has been operationalized) have led some theorists to argue that there is no unified account of evolvability to be had unless a particular level of description is prescribed (e.g., Houle and Pélabon 2023).^21^ Without losing information or applications, it is plausible that the notion might then be too vague (Pigliucci 2008). On the other hand, without explicitly identifying differing uses of the term, practitioners risk talking past one another (Brown 2014).

Our response and justification for denoting these components of our decomposition as ‘evolvability’ is, again, to emphasize that our proposal does not privilege a particular grain of description. In virtue of this feature, it provides the flexibility needed to account for these conflicting definitions—including the more controversial ones that have been offered—without losing grip on the concept entirely. Our view accommodates descriptions that stress the role of the organism, which emphasizes the capacity to generate variation via reproduction (e.g., Kirschner and Gerhart 1998). Using this definition of evolvability, the reference type might refer to the genetic architecture of a particular organism, for example. However, this same flexibility would also permit the reference type to be defined by a unique feature of a lineage such as the relaxation of a particular developmental constraint (Young et al. 2010). Similarly, definitions of evolvability that include populational features in the set of physical bases (e.g., Brown 2014; Sørensen 2011) can be accommodated by including these population-level features in the environment of reference. While we do not want to commit ourselves to the claim that this framework accommodates all that is ever meant by ‘evolvability’ in the literature, this serves to illustrate why our decomposition of *F* is not throwing arbitrary features of organisms or lineages into fitness, thus rendering it a grab bag. Rather, this proposal unifies different considerations about long-term evolutionary success.

This last point nonetheless brings us to another important remark: namely, that the definitions of evolvability *as property* need not correspond to the way in which evolvability is *operationalized*. For example, it is common to find that evolvability is predicated of a given *trait* when it is measured as the additive genetic variance of the trait in a given population (Houle 1992), that it might be associated with properties of the genotype–phenotype map in a given clade (Wagner and Altenberg 1996), or that it may be measured by the standing variation within a population rather than a population’s variability (Porto 2021; Wagner and Altenberg 1996). Mismatches between bearers and operationalizations are no more problematic than mismatches of the same kind when it comes to fitness: organismal-level properties are causally foundational, while their effects can be measured at multiple levels of interest.

Finally, we should dispel a further possible objection to our account. In proposing our framework, we have stressed the importance of typehood (with the organism and environment of reference) to distinguish different components of *F* and showed how doing so renders a more accurate picture of what evolutionary success amounts to. However, this might appear as though it is in tension with Pence and Ramsey’s reliance on the notion of organismal (or, more generally, token) fitness at the expense of trait (or type) fitness.^22^ However, we see no tension here. We have two responses. First, the propensity interpretation is only one possible way to interpret *F*. Other interpretations are possible (e.g., Abrams 2023, for an alternative interpretation), including one where *F* refers to a type/trait or where the distinction token/types evaporate since a type can be as complex as required to refer potentially to a single entity, as proposed in Takacs and Bourrat (2021, 2022). Therefore, whether or not one is a proponent of the PIF, the quadripartite decomposition of *F* is still relevant. Second, and more importantly, Pence and Ramsey’s targeting fitness *qua adaptedness* does not mean that typehood is not an important feature when considering dimensions of fitness different from adaptedness. Pence and Ramsey are even explicit about this point when they argue that ‘trait fitness … relies on individual fitness … but also includes factors such as heritability’ (2013, p. 872). We believe that Pence and Ramsey would agree with us that the ability to change type in a way that makes one better adapted is one such factor.^23^

## Conclusion

Building on the most sophisticated account of PIF in terms of *F* by Pence and Ramsey (2013), our framework offers valuable insights into the complexities of long-term evolutionary success by explicitly delineating two frames of reference—environment and type—that yield four components of long-term fitness: adaptedness, robustness of adaptedness, and two aspects of evolvability. This nuanced interpretation of *F* reconciles the explanatory gap between the classical idea of adaptedness as an ecological descriptor and long-term evolutionary success by making explicit that the former does not account exhaustively for the latter. One major contribution in the literature on evolvability has been to stress the importance of the variational factors for evolutionary outcomes in addition to ecological ones. Our account presents a comprehensive framework in which those factors are accounted for, and neither is discounted, as $V_{{E_{r} }}$ and $V_{{E_{ \sim r} }}$ can naturally be interpreted as evolvability.

In proposing our decomposition, we have steered clear of the mathematical assumptions anchoring Pence and Ramsey’s account. While some of these assumptions may appear at odds with the notion of evolvability, such as the assumption of weak ergodicity preventing any path dependence of evolutionary trajectories, we hope our account will stimulate new mathematical developments that accommodate evolvability considerations.

## Footnotes

1 Love (2003) labels these senses of evolvability ‘universal’ and ‘restricted,’ respectively.

2 Following Sober’s (2001) depictions of these two roles of fitness, other characterizations of this distinction have been proposed. Some have described the ecological role as ‘vernacular fitness’ (e.g., Matthen and Ariew 2002), or associated it with the capacity to solve ‘design problems’ of the environment (e.g., Dennett 1995). The mathematical role has also been characterized in different ways since its conception, including as strictly being ‘trait fitness’ (Walsh, Ariew, and Matthen 2017). See Pigliucci and Kaplan (2006) for an overview of the different usages.

3 For other examples, see Crow and Kimura (1956) and Takacs and Bourrat (2022).

4 PIF faces other difficulties. The ‘propensity’ part of PIF is a nod to the propensity interpretation of probability. This interpretation of probability theory comes with its own set of problems (e.g., Eagle 2004; Hájek 2019; Humphreys 1985) that have been further elucidated in the literature on PIF (e.g., Waters 1986; Sober 2013; Bourrat 2017). Here, we will focus on the objection that PIF does not provide a suitable bridge between the two roles of fitness.

5 We will consider here ‘trait fitness’ and ‘type fitness’ to be synonymous; however, see Abrams (2023, Chapter 3) for a subtler set of distinctions.

6 This proposal makes a number of assumptions, including ergodicity—there is no path dependence for this measure (Doulcier, Takacs, and Bourrat 2021; Bourrat 2023; Pence and Ramsey 2013, pp. 862–863). We will not discuss their other assumptions and, for now, will take ergodicity for granted. Our focus is to motivate their proposal from a conceptual rather than mathematical point of view.

7 Philosophical works in which the importance of the reference environment (or population) have been noted besides Brandon include Beatty (1984), Abrams (2009, 2014), Glymour (2014), Walsh (2010), and Takacs and Bourrat (2021).

8 Throughout, we use the term ‘possible daughter populations’ as shorthand for ‘possible daughter populations weighted by the probability that they are realized,’ as is done by Pence and Ramsey. Alternatively, we could stipulate (an empirically unrealistic level of) uniformity across the possible daughter populations, so that this feature of Pence and Ramsey’s view is not salient.

9 This case invites questions about what ‘type’ amounts to here. In Section “Decomposing *F*: Bridging the new PIF and evolvability”, we return to this question.

10 By, mutation, we meant something more abstract than point mutation. What we really meant is a change in type, where one type’s characteristic might be that it produces more variation than another type.

11 Notice that this is not only true when fitness is considered in the (very) long term; these features may be important considerations for fitness even on relatively short timescales.

12 This sense of robustness must not be conflated with other senses in the evolutionary literature and especially with the idea of genotypic robustness (i.e., persistence of phenotypes under genotypic changes) as associated with evolvability (e.g., how the term is used in Wagner 2008).

13 By convention, we will consider a (beneficial) change in type over the lifespan of an organism to be the result of phenotypic plasticity rather than evolvability. The reasoning here is that phenotypic plasticity does not typically involve genotypic changes, which are the ones that are considered evolutionarily relevant. However, depending on the scale of the type of reference, phenotypic plasticity could be seen as evolvability and vice versa. For instance, in cases of organisms with very long lifespan such as the quaking aspen, where differences in fitness amounts to differences in persistence rather than reproductive output (see Bouchard 2008, 2011; Bourrat 2014; Papale 2021), considering changes in type during the lifetime of the organism becomes evolutionarily significant. Conversely, organisms that are able to mutate rapidly as part of their evolutionary strategy, such as the hypermutator strains of *Pseudomonas aeruginosa* involved in chronic lung infections (Maciá et al. 2005), despite changing their type (i.e., their genotype) would nonetheless be considered as a single type when compared to non-hypermutator strains. For a discussion of the relationship between phenotypic plasticity and evolvability, see West-Eberhard (2003).

14 Abrams (2023) seems to be on the same track when he provides counterexamples to Pence and Ramsey’s view.

15 Our account also inherits a feature of Pence and Ramsey’s account that might be regarded as a problem. *F* requires knowing the probability distribution of *all possible* events affecting the survival and reproduction of the entities and its descent. What is possible or not possible might be regarded as too vague to be well defined. However, note that Pence and Ramsey’s account (and consequently ours) will be compatible with any operationalization of what events are regarded as possible and their probability.

16 Although we focus here on evolvability, we believe a similar thing could be said about robustness of adaptedness.

17 This is also why evolvability is sometimes predicated on lineages (Brown 2014; Nuño De La Rosa and Villegas 2022; Sterelny 2007).

18 It also has some similarities with the reference class problem in the philosophy of probability (Bourrat 2020; Hájek 2019).

19 As they effectively do when presenting their account (Pence and Ramsey 2013, pp. 860–861).

20 See Villegas (2024) for a recent discussion of this separation in the context of the debate on ultimate and proximate causes.

21 For example, quantitative geneticists usually define it as the ‘the ability of a population to respond to selection’ (Houle 1992, p. 195), while some evo-devo biologists refer to it as ‘an organism’s capacity to generate heritable phenotypic variation’ (Kirschner and Gerhart 1998, p. 8420). Other definitions include reference to the developmental system (Hendrikse et al. 2007), or the genotype-phenotype map (Hansen 2006).

22 This distinction has been at the center of a debate about whether the crucial sense of fitness for the study of evolution is fitness at the organismal level (e.g., Pence and Ramsey 2013, 2015) or at the trait or population level (Abrams 2012; Sober 2013).

23 Note, in reference to the Pence and Ramsey quotation, that the quantitative genetics sense of evolvability is closely tied to the notion of heritability (see Hansen, Pélabon, and Houle 2011, for a discussion of the relationship between heritability and evolvability as used in quantitative genetics).
